# Transforming growth factor beta 1 induces methylation changes in lung fibroblasts

**DOI:** 10.1371/journal.pone.0223512

**Published:** 2019-10-11

**Authors:** Miguel Negreros, James S. Hagood, Celia R. Espinoza, Yalbi I. Balderas-Martínez, Moisés Selman, Annie Pardo

**Affiliations:** 1 Facultad de Ciencias Universidad Nacional Autónoma de México, Mexico City, Mexico; 2 Department of Pediatrics, Division of Respiratory Medicine, University of California-San Diego, La Jolla, California, United States of America; 3 Department of Pediatrics, Pulmonology Division, University of North Carolina at Chapel Hill, Chapel Hill, North Carolina, United States of America; 4 Instituto Nacional de Enfermedades Respiratorias Ismael Cosío Villegas, Mexico City, Mexico; 5 Cátedra CONACyT-INER, Mexico City, Mexico; Shantou University, CHINA

## Abstract

Idiopathic pulmonary fibrosis is a complex disease of unknown etiology. Environmental factors can affect disease susceptibility via epigenetic effects. Few studies explore global DNA methylation in lung fibroblasts, but none have focused on transforming growth factor beta-1 (TGF-β1) as a potential modifier of the DNA methylome. Here we analyzed changes in methylation and gene transcription in normal and IPF fibroblasts following TGF-β1 treatment. We analyzed the effects of TGF-β1 on primary fibroblasts derived from normal or IPF lungs treated for 24 hours and 5 days using the Illumina 450k Human Methylation array and the Prime View Human Gene Expression Array. TGF-β1 induced an increased number of gene expression changes after short term treatment in normal fibroblasts, whereas greater methylation changes were observed following long term stimulation mainly in IPF fibroblasts. DNA methyltransferase 3 alpha (DMNT3a) and tet methylcytosine dioxygenase 3 (TET3) were upregulated after 5-days TGF-β1 treatment in both cell types, whereas DNMT3a was upregulated after 24h only in IPF fibroblasts. Our findings demonstrate that TGF-β1 induced the upregulation of DNMT3a and TET3 expression and profound changes in the DNA methylation pattern of fibroblasts, mainly in those derived from IPF lungs.

## Introduction

Idiopathic Pulmonary Fibrosis (IPF) is an irreversible, chronic, progressive and lethal disease of unknown etiology, characterized by excessive extracellular matrix (ECM) deposition [1]. ECM is secreted mainly by activated fibroblasts under certain stimuli [2].

Several molecules/cytokines have been previously described as key effectors of the fibrotic response. Among them, transforming growth factor-beta 1(TGF-β1) is considered one of the most potent mediators of tissue remodeling and fibrosis [3, 4]. TGF-β1 promotes fibroblast proliferation, and their differentiation to myofibroblasts, highly synthetic cells producing multiple ECM components (mainly collagens) with increased contractility and apoptosis resistance [5–8]. This phenotypic change is the result of the strong effect of TGF-β1 on global gene expression [9, 10], likely associated with epigenetic mechanisms [11]. Additionally, recent studies have linked alterations in DNA methylation, one of the best understood epigenetic processes, with aging, a driver of IPF [12].

Some prior studies have analyzed DNA methylation differences in IPF and normal human lungs, but studies on isolated fibroblasts and those evaluating primarily the role of TGF-β1 are scanty [13, 14]. One targeted study considers TGF-β1 as a DNA methylation modulator; however, it lacks analysis of the global methylation landscape [15].

In our study, we used Illumina 450k technology to evaluate methylation of 485,512 CpG sites, in normal and IPF derived fibroblasts under short (24h) and longer-term (5d) TGF-β1 stimulation. In addition, we examined the methylation relative to gene location and the relationship to changes in gene expression.

## Materials and methods

### Cell culture

Primary human lung fibroblasts were obtained from normal and IPF lungs under protocols approved by the ethics committee of the Instituto Nacional de Enfermedades Respiratorias (INER), and the participants gave written informed consent. IPF fibroblasts were obtained from five patients by open lung biopsy usually performed 1 week after hospital admission for diagnosis purposes. IPF was diagnosed as described elsewhere, including the characteristic high-resolution computed tomography imaging and morphology of usual interstitial pneumonia [16] Interstitial lung diseases associated with connective tissue disorders or with environmental and occupational exposures were excluded.

Patients (three male and two female; four former smokers and one nonsmoker) aged 63 ± 5 yr (mean ± standard deviation [SD]) were inpatients at INER, Mexico City. All of them had progressive dyspnea, restrictive pulmonary function tests (forced vital capacity: 67 ± 3%), reduced diffusion lung capacity of carbon monoxide (DLCO: 59.2 ± 3.5%), and hypoxemia at rest (91 ± 1.5%) worsening with exercise (81 ± 3.5%). None of the patients had been treated with corticosteroids or immunosuppressive drugs at the time of biopsy. [17]

Control fibroblasts (n = 4; three male and one female; three smokers and one nonsmoker) were derived from lungs of age-matched patients undergoing lobectomy/pneumonectomy for removal of a primary lung tumor which showed no histologic evidence of disease. Cells were obtained by enzymatic dispersion with trypsin (Sigma-Aldrich). Additionally, a normal human lung fibroblast (NHLF) cell line was obtained from Lonza. All primary cultures were grown in Ham’s F-12 medium (Gibco) with 10% FBS (Gibco), while NHLF were grown in Lonza FGM, at 37°C in an atmosphere of 95% air and 5% CO_2_ until early confluence from passage 6 to 8. Then, cells were treated with TGF-β1 (10 ng/ml), using two different time periods (24 hours or 5 days) all of them cultured in Ham’s F-12 with low serum (0.1%). The dosage and time periods were determined based on the results of previous studies [18].

### DNA extraction

Genomic DNA extraction was performed with MasterPure Complete DNA and RNA purification Kit (Epicentre) following the manufacturer’s instructions. Total DNA was resuspended in a volume of 20μl. DNA concentration and quality were determined by spectrophotometry (ND-1000 UV/Vis Spectrophotometer, NanoDropTecnologies USA).

### RNA extraction and qRT-PCR analysis

Total RNA was extracted from human lung fibroblasts with TRIzol reagent (Invitrogen), according to the manufacturer’s instructions. 1μg of RNA was reverse transcribed into cDNA, and real-time PCR was performed using Verso cDNA Synthesis Kit (Applied Biosystems) according to the manufacturer’s instructions.

Fibroblast gene expression screening analysis for collagen type I alpha 1 chain (COL1A1), collagen type I alpha 2 chain (COL1A2), actin alpha 2 smooth muscle (ACTA2), Thy-1 cell surface antigen (THY1), fibronectin containing extra domain A (FnEDA), and hypoxanthine phosphoribosyltransferase 1 (HPRT1; as housekeeping gene for normalization) were performed with SYBR Green PCR Master Mix (BioRad) (**S1 Table**).

DNA methyltransferase 3 alpha (DNMT3a) and tet methylcytosine dioxygenase 3 (TET3) expression were determined by real-time PCR using TaqMan Gene Expression Assays (Applied Biosystems) and RNA polymerase II subunit A (POLR2A) was used as housekeeping gene for normalization. The amplification reactions were done in a LightCycler 480 (Roche) with Maxima Probe qPCR Master Mix (Thermo Fisher Scientific). The relative quantitation method based on the 2 ^-ΔCT^ was used to analyze the results of two independent experiments made in triplicate.

### Global gene expression and DNA methylation assays

Global RNA assay was performed with Affymetrix GeneChip PrimeViewT Human Gene Expression Array. The array was processed using R 3.4.2 [19], using Bioconductor 3.6 [20], with affy package. Background correction and data normalization were performed using RMA. 49,495 probes were calculated for their expression level. Expression levels of three technical replicates of each condition were averaged. To obtain differentially expressed genes, Limma package was used [21]. Probes with adjusted *p*-value < 0.01 and log2 fold change > 2 and < -2 were considered as differentially expressed and were mapped to the genome using Affymetrix annotation.

For the DNA methylation global assay, Infinium Human Methylation 450 BeadChip kit was used as per manufacturer instructions. Data were analyzed using R 3.4.2 [19], using Bioconductor 3.6 [20], following the pipeline of Maksimovic [22]. Methylation status of 485,512 loci was determined [23]. For all probed genomic positions, detection *p*-values >0.01 were filtered out (485,179 loci). Probes with SNPs at CpG or SBE site were filtered (456,352), followed by filtering of probes that have shown to be cross-reactive, leaving 429,792 probes used for analysis [24]. For differential expression analysis, the methylation levels of two replicates under each condition were averaged to consider a region as a differentially methylated locus (DML) and selected according to limma package results (*p*-value < 0.00005). CpG probes were associated with RefSeq genes, island regions and subclassified according to the UCSC annotation database [25]. For the analysis, gene regions were grouped as follows: Promoter (TSS1500 and TSS200), 5’UTR/1^st^ Exon, Body and 3’ UTR. Overlapped genes from methylation and expression were collected.

The high-throughput data was submitted to the Gene Expression Omnibus database (https://www.ncbi.nlm.nih.gov/geo/), SuperSerie accession number: GSE135099 (Expression data: GSE135065, Methylation data: GSE135097).

### Enrichment analysis

To analyze the biological significance of microarray expression changes, significant up- and down- regulated genes that passed adjusted *p*-value < 0.01 were uploaded to Enrichr tool to perform enrichment analysis with gene ontology biological processes (GOBP) [26].

GO terms that passed adjusted *p*-value <0.01 were ordered using combined score. Categories that shared the same genes were discarded to eliminate group overrepresentation. The top 10 GOBP terms were chosen for graphs. The detailed gene lists and GO/ analysis are attached separately (**S2 Table**).

## Results

### Fibroblasts’ response to TGF-β1

**Fig 1:**
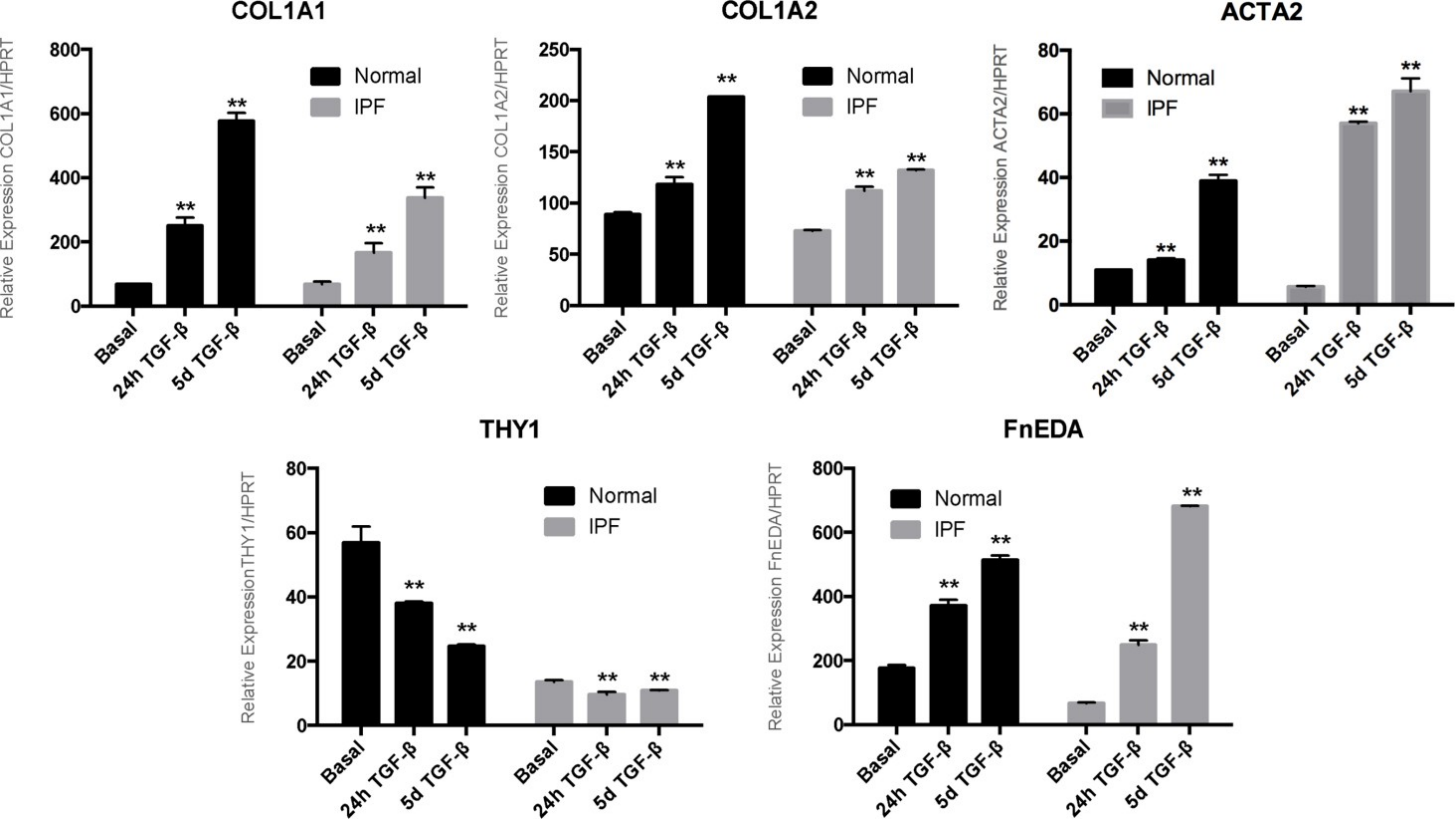
qRT-PCR screening analysis of normal and IPF fibroblast cell lines after TGF-β1 stimuli. Dark bars represent normal fibroblast and grey bars their IPF counterpart gene expression of collagens (COL1A1 and COL1A2), EDA fibronectin (FnEDA) α-smooth muscle actin (ACTA2) and Thy-1 (THY1), respectively These are the result of two independent experiments; **p<0.05.

From a group of four primary cell lines examined from normal human lungs and five from IPF lungs, we chose one of each group to evaluate the global gene expression and methylation. We based our selection on a qRT-PCR screening of genes with well-known response to TGF-β1 [9, 15, 27–29]. Both selected fibroblast cell lines showed significantly increased expression of COL1A1, COL1A2, ACTA2, FnEDA after 24-hours and 5-days of TGF-β1 treatment. A significantly decreased expression of THY1 after 24 hours and 5 days of TGF-β1 treatment was observed, although the IPF line selected had lower basal THY1 expression, as we have demonstrated previously [30–32]. (**Fig 1**)

Interestingly, after 5 days of TGF-β1 treatment IPF fibroblasts showed a higher expression of ACTA2, and FnEDA and a lower increase of COL1A1 and A2 compared with the normal ones.

### TGF-β1-induced gene expression changes

**Fig 2:**
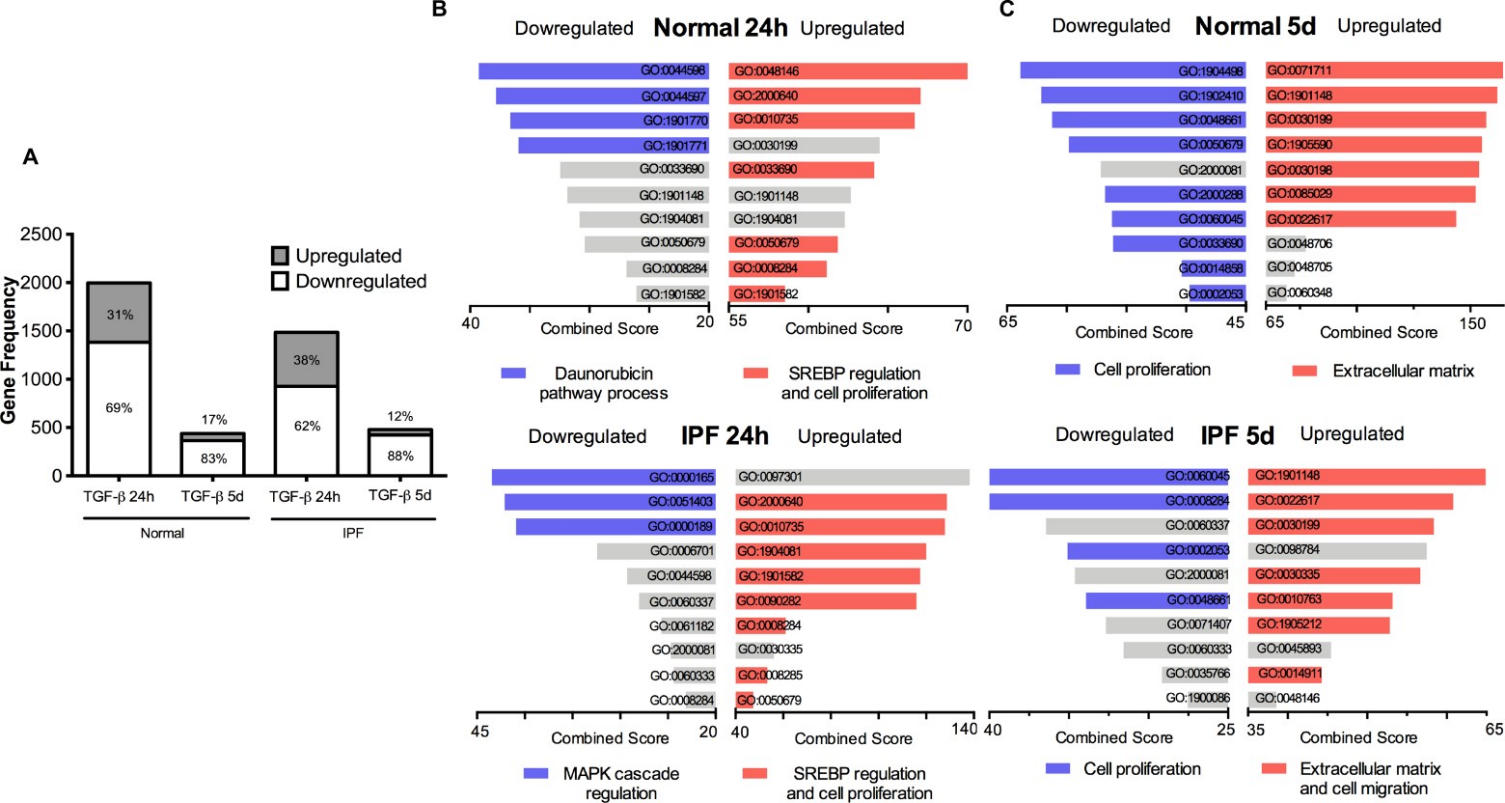
TGF-β1 changed gene expression predominantly at 24 hours and GOBP category enrichment differed between treatments. Number of genes that changed expression after 24 hours (24h) or 5 days (5d) of TGF-β1 treatment in normal or IPF derived fibroblasts. The number inside or over the white (downregulation) or gray (upregulation) bars represent the percentage of genes that present expression changes (A). Groups of bars represent the top ten GOBP terms ranked by combined score, associated with short-term (B) and long-term (C) treatment. Each graph groups GOs of downregulated genes in the left and upregulated genes on the right. Terms belonging to closely related categories are highlighted in the same color (blue for downregulated and red for upregulated genes) as specified in each graph. Grey bars represent ungrouped categories, each bar details are specified in (**S2 Table**).

Microarray expression analysis revealed that a higher number of genes had expression changes after short-term (24 hours), compared with long-term (5 days) TGF-β1 stimulus. Likewise, short-term treatment induced greater changes in normal fibroblasts (25%) than in IPF fibroblasts (**S3–S6 Tables).** As shown in **Fig 2A**, in both IPF and normal cell lines a greater proportion of genes were downregulated, which even was more evident at 5 five days.

In order to compare if the effect of the TGF-β1 stimuli was similar or different in fibroblasts derived from normal or IPF lungs, we performed a Gene Ontology enrichment analysis of Biological Processes (GOBP) with the significantly up- and down-modulated genes of the Array. Genes upregulated with short-term TGF-β1 treatment revealed enrichment in categories related to sterol regulatory element-binding protein (SREBP) signaling and regulation of cell proliferation in both fibroblast lines. In contrast, the enriched groups for the downregulated genes were different. In the normal cell line, changes were related to signaling associated with the anti-tumor drug, daunorubicin, while the changes in the IPF derived fibroblasts were associated with Mitogen-Activated Protein Kinases (MAPK) processes (**Fig 2B**).

On the other hand, the upregulated genes with long-term TGF-β1 treatment comprised categories associated with extracellular matrix production in both cell lines; additionally, the IPF line showed enrichment for the cell migration category. Also, the down-modulated group of genes in both fibroblast lines were enriched for cell proliferation categories (**Fig 2C**).

### Effect of TGF-β1 on the methylation machinery

**Fig 3:**
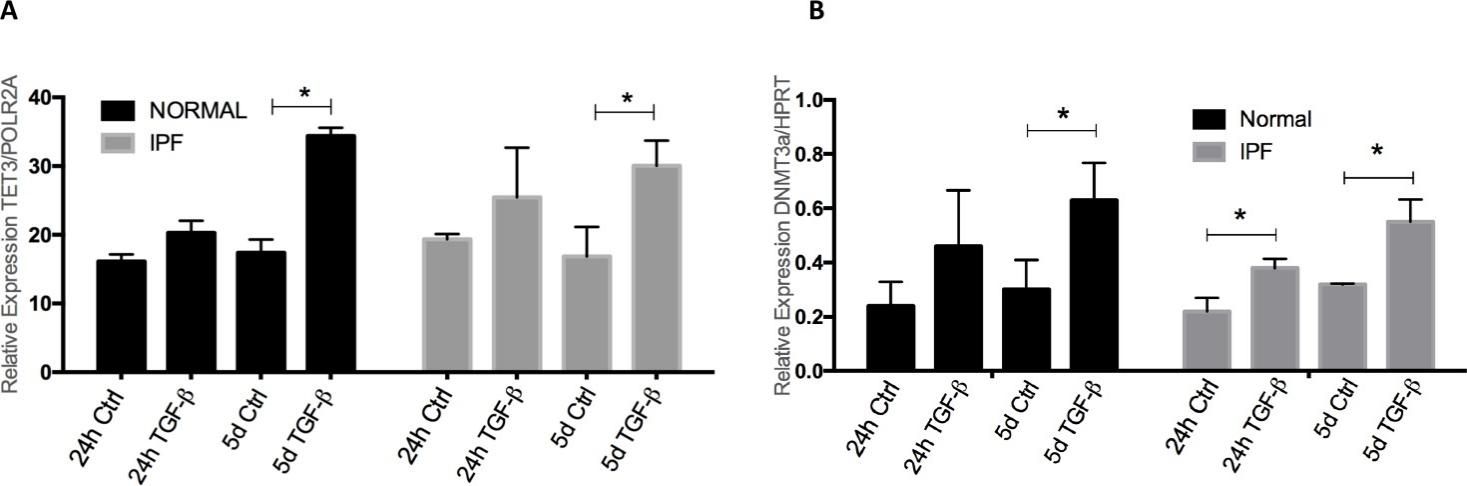
TGF-β1 alters the expression of the methylation/demethylation machinery. Black and Gray bars represent normal and IPF fibroblasts, respectively. qRT-PCR of TET3 (A) and DNMT3a (B) after short and long term TGF-β treatment. (*p<0.05) The figure shows one representative cell line of each group from two normal and two IPF cell lines tested. All measurements are result of two independent triplicate experiments.

Microarray expression analysis showed that Tet methylcytosine dioxygenase 3 (TET3) was upregulated in both normal and IPF fibroblast cell lines. To confirm this finding, TET3 was measured by real-time PCR in two IPF and two normal cell lines, at 1 and five days of stimulus with TGF-β1. As shown in **Fig 3A**, while no changes were observed at 1 day, at 5 days TGF-β1 induced a significant increase of TET3. Recently, TET family proteins have been described as part of the cytosine demethylation process. Since methylation is dynamically regulated through the coordinated action of the DNMTs and TET enzymes, we wondered whether the expression of DNMTs was also modified by TGF-β1. We examined DNMT1, DNMT3a and DNMT3b (DNA methyltransferase 1, 3 alpha and 3 beta), and we found that DNMT3a was significantly upregulated after 24 hours in IPF fibroblast and after 5-day treatment in both cell lines (**Fig 3B**, *p*<0.05).

### Effect of TGF-β1 on the methylome

**Fig 4:**
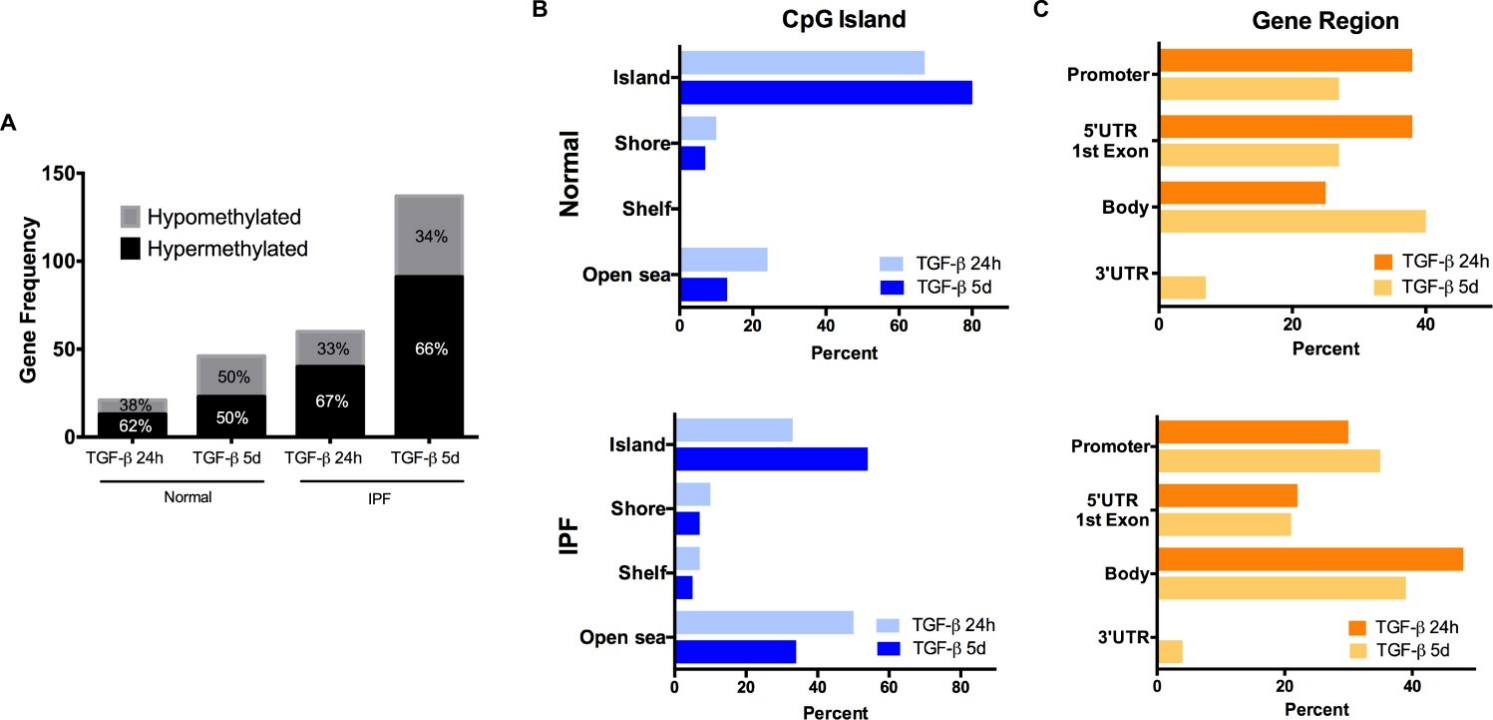
Methylation gene frequency and gene localization/ CpG density of those changes after TGF-β1 treatment. (A) The number of genes with changed methylation status in normal and IPF fibroblast after 24 hours (24h) or five days (5d) of TGF-β1 treatment. Numbers inside the bars represent the percentage of hypomethylated (gray), hypermethylated (black) genes. (Fold Change) (B) Percentage of genes that change methylation status relative to CpG island distance (Island, Shore, Shelf and Open sea) or to the Gene (Promoter, 5’UTR/1^st^ Exon, Body and 3’UTR) at 24 hours (light blue, orange) and five days (dark blue, yellow) in Normal (upper) and IPF (lower) fibroblasts.

TGF-β1 modified DNA methylation of normal and IPF fibroblasts after 24 hours and 5 days of TGF-β1 stimulus (**Fig 4A**). In both cell lines the effect was significantly higher at 5 days. Another remarkable feature was the presence of more gene methylation changes in the IPF fibroblasts in comparison with the normal cell line with the majority of the changes representing hypermethylation. (**S7–S10 Tables, Fig 4A**).

Despite DNA methylation having been studied mostly in gene promoters and in stretches of CpG dinucleotides (known as Islands), this modification can occur anywhere in the gene and more distant from these clusters. The impact on gene expression depends in part on both CpG-density and gene localization where DNA methylation occurs [33].

When we focused on the distribution of these changes in relation to the CpG Island distance, we found that in the normal fibroblasts most methylation changes induced by TGF-β1 occurred directly in the islands (67–80%), whereas in the IPF fibroblasts, the Island and Open sea were the best represented groups (between 33–54% and 50–34%, respectively; **Fig 4B**).

On the other hand, when the methylation changes were analyzed in relation to the UCSC database gene regions (22), most of the changes in the normal fibroblasts are similarly distributed in three categories: Promoter, 5’UTR/1^st^Exon, and Body (~30%), although the first two seem to decrease with sustained TGF-β1 treatment. In the IPF cell line, the most notable characteristic was the overrepresentation of the gene body category (~40%) and, unlike the case in normal fibroblasts, it decreased with the treatment time. (**Fig 4C**)

### Overlap between gene methylation and gene expression

**Fig 5:**
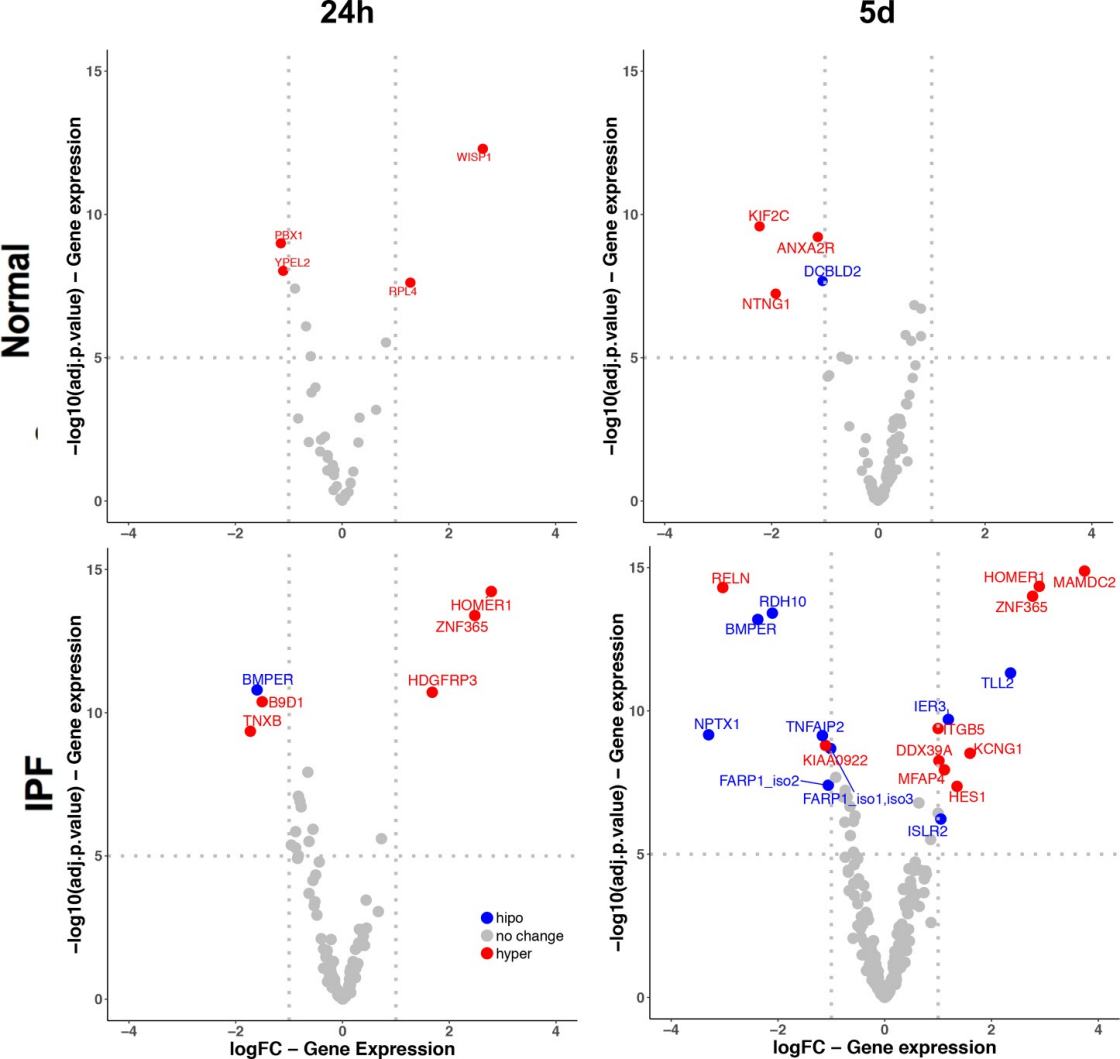
Volcano plots of differentially expressed and methylated genes following TGF-β1 stimuli. Vertical lines indicate the threshold for fold change (logFC) of -1 or 1-fold compared to controls. The horizontal line represents the threshold of a 5 -log10 P value. The blue and red points represent hypo and hypermethylated genes, grey points represent genes with no significant change. Some genes can be duplicated if more than one probe is associated with a modulated gene.

Few genes exhibited an overlap of methylation and expression changes. Unlike other studies, we did not restrict our analysis to those with an inverse correlation (**S1 Fig**). In fibroblasts from normal human lungs, a limited number of genes changed and hypermethylation was prevalent. In the case of the IPF fibroblasts we found under extended treatment (5 days) the largest number of genes with altered expression levels and the highest rates of hypomethylation (**Fig 5**)

**Fig 6:**
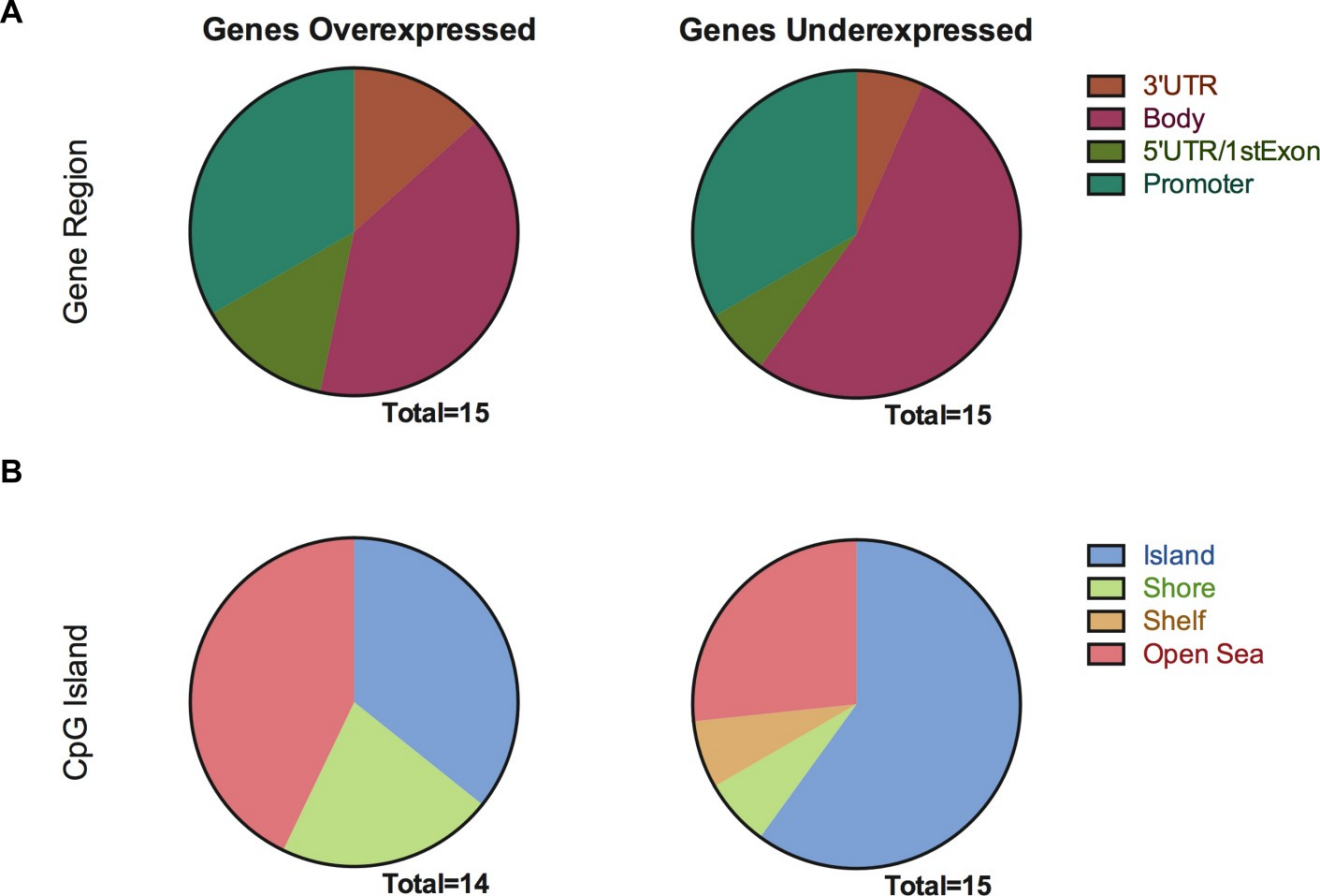
DNA Methylation changes gene-localization or CpG-density of genes that also changed expression, after TGF-β1 treatments. Pie charts represent all the genes that change their states of DNA methylation and gene expression (over and underexpressed, left and right, respectively) after TGF-β1 treatment; the methylation is arranged by UCSC gene regions (upper) and in relation to the distance from CpG islands (lower).

The genes that share changes in methylation and gene expression after TGF-β1 treatment were very limited. In this context, we analyzed all the groups together to find out a possible pattern independently of a positive or inverse correlation. When methylation was classified by gene region, we didn’t find any difference between groups of expressed genes (**Fig 6A**), but when we focus on CpG Island distance, we found that downregulated genes are associated with methylation that occurs directly in the CpG Island while Open Sea is best represented category in the overexpressed genes. (**Fig 6B**)

## Discussion

During repair following tissue damage, lung fibroblasts must coordinately express and silence genes involved in processes such as migration, proliferation, deposition of extracellular matrix, differentiation and apoptosis, which will allow the correct repair of the insult. A complex interplay of epigenetic mechanisms coordinate the correct execution of those programs, and the failure of this regulation has been associated with factors such as age and environment [11]. The loss of this coordination, as occurs in IPF, will lead to pathological phenotypes associated with the disease (1, 28).

TGF-β1 is likely the strongest profibrotic mediator and plays a critical role in the activation of fibroblasts, but its effects on DNA methylation are not well described. In the present study, we focused on the effects of short and longer-term exposure to TGF-β1 on gene expression and DNA methylation patterns, as well as changes in regulators of DNA methylation, in IPF and normal lung fibroblasts. We found that a 24h TGF-β1 stimulus generates gene expression changes related to regulatory pathways and transcriptional factors (MAPK, SREBP, and daunorubicin-associated pathways) while the longer-term stimulus provoked changes related to the fibrotic phenotype of fibroblasts, previously described in IPF, such as increased migration and secretion of matrix molecules and decreased proliferation [34]. Additionally, we found that TGF-β1 generates a higher number of changes in expression at 24 hours, in contrast to changes in methylation that mainly occur at five days. Interestingly, normal fibroblasts showed significantly more gene expression changes, while IPF fibroblasts underwent more modifications in the methylome. The changes in methylation might be related to the increased DNMT3a expression that we found after TGF-β1 stimulation. Interestingly, in renal fibrosis, it has been reported that long term TGF-β1 stimulation induced DNA methylation, but DNMT1 was found to be related to this change. [18].

Use of the Illumina 450k platform allowed us to determine the exact localization of methylation changes with respect to genes and CpG islands, which in turn, enabled us to discover that after TGF-β1 treatment, most DNA methylation changes occurred in the gene body, confirming previously reported findings in fibrotic lungs [35]. Furthermore, when we analyzed the group of genes that change expression, in addition to undergoing changes in DNA methylation, we again found that these changes were primarily found in the gene body, supporting the notion that although gene expression changes have been mainly associated with methylation in the promoter region, the gene body plays an important role and its methylation is related to gene overexpression, regulation of isoforms, nucleosome stability and transcription efficiency as found in genome-wide methylation studies [36–38].

Our data shows that most of the TGF-beta- associated methylation changes occur in the islands and very few in the surrounding regions such as CpG shores and shelves, which differs from the findings previously reported in whole lungs [35]. Our study differs from the latter in that a different platform was used, and the techniques have different intrinsic biases (e. g. changes are limited to regions in proximity to enzymes recognition sites in the CHARM method, whereas the Infinium Methylation platform coverage is dependent on assay design) [39]. However, our study reports significant changes in DNA methylation in CpG islands, mainly in genes with downregulated expression; further research will be needed to decipher the biological significance of these changes.

As in other previous studies using either whole lungs or isolated fibroblasts, we did not find a correlation pattern in global methylation that accounts for all the changes of gene expression related to TGF-β1 [13, 14, 35]. In whole-lung studies, it has been suggested that the diversity and the variable number of cell types in the lung may explain the lack of overlap. However, even in a prior study using lung fibroblasts, there was a lack of correlation reported [40]. Heterogeneity of the lung fibroblasts, as well as the existence of other epigenetic mechanisms regulated by TGF-β1, such as altered expression of the histone methyltransferase (SET and MYND domain containing 3: SMYD3) [41], which we found overexpressed in our study (S3, S4, S5 and S6 Tables), may contribute to the lack of correlation.

In addition, we also found overexpression of TET3, a member of the TET family responsible for hydroxymethylation and subsequent demethylation on DNA. Hydroxymethylation can’t be distinguished from classical methylation with bisulfite approaches, and this modification has not been methodologically addressed by global studies of methylation in IPF [42]. The relative roles of TET3 and DNMT3a on the expression of any given gene cannot be determined from our findings.

The most important limitation of our study is the number of samples used; however, our focus in this study is more on the mechanisms of the response to TGF-β1. Future research should include greater number of cell lines to circumvent inter-individual bias, as methodological strategies to study DNA methylation across the whole genome, including analysis of hydroxymethylation.

Two interesting and novel findings resulted from the gene ontology analysis: the identification of modification of pathways related to SREBP and daunorubicin. Those molecules have been extensively studied and related to lipid homeostasis and to antitumor therapy, respectively. SREBP has been studied in lung injury responses on other cell types such as alveolar Type II [43, 44] cells, and has been implicated in fibrosis in other organs such as kidney and liver [45, 46]. In this regard, findings from a study made in renal fibrosis found that inhibition of SREBP can diminish extracellular matrix deposition due fibroblast activation [47]. Daunorubicin has been associated in the development of myocardial fibrosis, but this is the first time it has been related to lung fibroblasts and possible regulation of a fibrotic phenotype [48]. One study reported that daunorubicin could prevent the formation of epineural fibrosis by inhibition of fibroblast proliferation [49]. Altogether these findings might suggest a possible role for therapeutic targeting of these pathways, however, more studies are needed.

## Conclusions

In summary, we have examined for the first time the effect of TGF-β1 on DNA methylation in lung fibroblasts. We found that it affects some important elements of the DNA methylation/demethylation machinery (DNMT3a and TET3) and that IPF versus normal lung derived fibroblasts respond differently, likely because of the prior exposure to the fibrotic environment within the lung.

## Supporting information

S1 Fig Details of genes with overlapping methylation and expression changes followng TGF-β1 stimulation. Bars and diamonds represent gene expression and methylation changes against controls. The location of the methylation (related to CpG island or to gene compartment) is shown in the boxes below.
(TIF)

S1 Table Primers for qPCR analysis. (XLSX)

S2 Table Gene ontology (GO) annotation. (XLSX)

S3 Table Microarray—Normal TGF-beta 24 hrs vs normal control. (XLSX)

S4 Table Microarray—Normal TGF-beta 5 days vs normal control. (XLSX)

S5 Table Microarray—IPF TGF-beta 24 hrs vs IPF control. (XLSX)

S6 Table Microarray—IPF TGF-beta 5 days vs IPF control. (XLSX)

S7 Table Methylation array—Normal TGF-beta 24 hrs vs normal control. (XLSX)

S8 Table Methylation array—Normal TGF-beta 5 days vs normal TGF-beta control. (XLSX)

S9 Table Methylation array—IPF TGF-beta 24 hrs vs IPF control. (XLSX)

S10 Table Methylation array—IPF TGF-beta 5 days vs IPF control. (XLSX)
