# Correction: Transforming growth factor beta 1 induces methylation changes in lung fibroblasts

**DOI:** 10.1371/journal.pone.0224841

**Published:** 2019-10-30

**Authors:** The PLOS ONE Staff

The following information is missing from the Funding statement: MN is a doctoral student from Programa de Doctorado en Ciencias Biomédicas, Universidad Nacional Autónoma de México (UNAM), and received fellowship 239706 from Consejo Nacional de Ciencia y Tecnología, México (CONACyT) to support this study. The publisher apologizes for the error.
