# Supplementary figures and images for: Transforming growth factor beta 1 induces methylation changes in lung fibroblasts
Source: PLoS One. 2019 Oct 11;14(10):e0223512. doi: 10.1371/journal.pone.0223512 (PMC6788707; doi:10.1371/journal.pone.0223512)

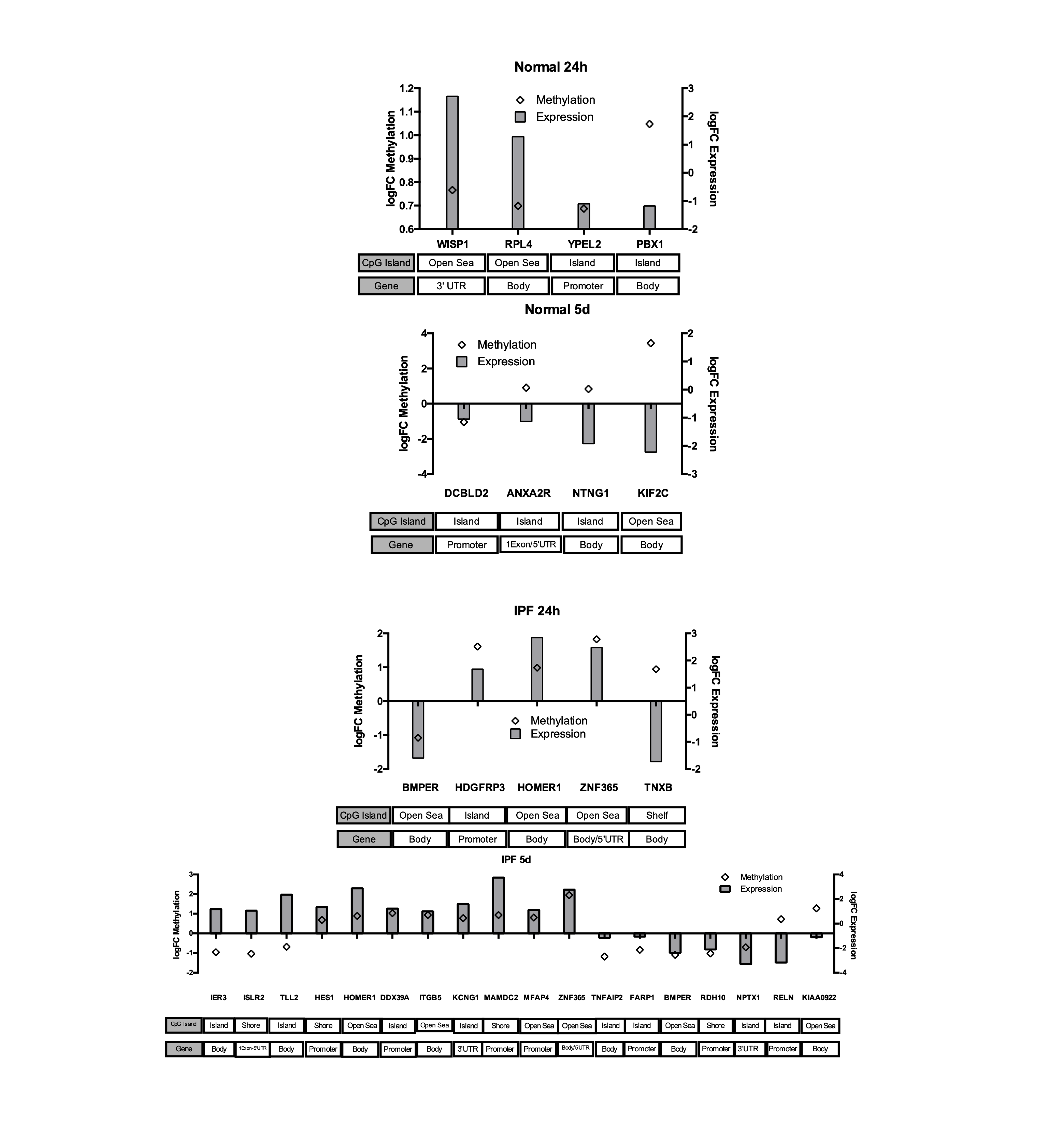

Supplement: S1 Fig — Bars and diamonds represent gene expression and methylation changes against controls. The location of the methylation (related to CpG island or to gene compartment) is shown in the boxes below. (TIF) [file pone.0223512.s001.tif]
